# Open-Face Masks in Radiotherapy: Enhancing Therapeutic Strategies for Head and Neck and Brain Cancer Patients—A Comprehensive Scoping Review

**DOI:** 10.3390/cancers16162899

**Published:** 2024-08-21

**Authors:** Andrea Lastrucci, Ilaria Morelli, Claudio Votta, Irene Maran, Nicola Iosca, Ilaria Pia Monaco, Viola Salvestrini, Isacco Desideri, Livia Marrazzo, Yannick Wandael, Patrizia Cornacchione, Stefania Pallotta, Daniele Giansanti, Renzo Ricci, Lorenzo Livi, Pierluigi Bonomo

**Affiliations:** 1 Department of Allied Health Professions, Azienda Ospedaliero-Universitaria Careggi, 50134 Florence, Italy; 2 Department of Experimental and Clinical Biomedical Sciences “M. Serio”, University of Florence, 50134 Florence, Italylivia.marrazzo@unifi.it (L.M.);; 3 Dipartimento Diagnostica per Immagini e Radioterapia Oncologica, Fondazione Policlinico Universitario A. Gemelli IRCCS, 00168 Roma, Italy; 4 Radiation Oncology, IRCCS Azienda Ospedaliero-Universitaria di Bologna, 40138 Bologna, Italy; 5 Radiation Oncology, Department of Diagnostic Pathology, Bioimages and Public Health, Azienda Ospedaliero-Universitaria Consorziale Policlinico, 70120 Bari, Italy; 6 Radiation Oncology Unit, Azienda Ospedaliero-Universitaria Careggi, 50134 Florence, Italy; 7 Medical Physics Unit, Careggi University Hospital, 50134 Florence, Italy; 8 Centre TISP, Istituto Superiore di Sanità, 00161 Rome, Italy; daniele.giansanti@iss.it

**Keywords:** brain and head-and-neck cancer, open-face mask, discomfort, interfraction variability, intrafraction variability

## Abstract

**Simple Summary:**

Conventional closed face masks (CFMs) are used in radiotherapy for head and neck cancer (HNC) and brain cancer (BC) but can cause discomfort and anxiety, affecting the patient experience. Recently, open-face masks (OFMs) have been introduced as an alternative. This study reviews and explores the application and use of OFMs in the treatment of HNC and BC, analyzing 19 relevant studies. The analysis shows heterogeneity in the type of OFMs used, especially in BC. In some cases, they are used alone, while in others, they are combined with complementary devices such as mouth bites. For both treatment sites, the review shows that OFMs, especially in combination with surface-guided radiotherapy (SGRT), offer significant advantages in terms of patient comfort and positioning accuracy. The results suggest that OFMs can achieve sub-millimeter and sub-degree reproducibility, which supports their clinical integration.

**Abstract:**

**Introduction**: The main goal of radiotherapy (RT) is to deliver a precise dose to the target while sparing the surrounding normal tissue and minimizing side effects. Appropriate patient immobilization is crucial, especially for head and neck cancer (HNC) and Brain Cancer (BC). Conventional closed-face masks (CFMs), while effective in minimizing head motion, can cause significant discomfort, anxiety, and claustrophobia. Open-face masks (OFMs) have been developed to increase patient comfort while providing precise immobilization. **Methods:** Following the Preferred Reporting Items for Systematic Review and Meta-Analysis (PRISMA) extension for scoping reviews and the Arskey and O’Malley framework, an electronic search of EMBASE, PubMed, SCOPUS, and Web of Science was conducted to identify original studies reporting the use and description of OFMs in clinical practice up to April 2024. The inclusion criteria were English-language articles focusing on OFMs for HNC and BC patients undergoing RT. **Results:** Of 618 titles, 19 articles fulfilled the selection criteria. Most studies were comparative (*n* = 13) or observational (*n* = 6). The articles were categorized by treatment site, resulting in three groups: BC (*n* = 14, 68.4%), HNC (*n* = 4, 21.4%), and mixed (*n* = 2, 10.5%), which includes both BC and HNC. Of note, 82.4% (*n* = 16) of the included studies were published from 2020 onwards, emphasizing the recent adoption of OFM in clinical practice. **Conclusions**: The reviewed studies show that OFMs, in combination with SGRT, offer significant advantages in terms of patient comfort and positioning accuracy in HNC and BC treatments. Reproducibility in the sub-millimeter and sub-degree range can be achieved, which supports the use of OFMs in clinical practice. Future research should explore innovative combinations of immobilization and monitoring to further improve RT outcomes and ensure precise treatment while increasing patient comfort.

## 1. Introduction

The main goal of radiation therapy (RT) is to deliver a precise dose to the target while sparing surrounding normal tissue, thus minimizing side effects. Therefore, the appropriate immobilization of patients plays a key factor in the RT workflow [1], even if patients undergoing RT frequently experience significant discomfort when immobilization techniques are adopted. Particularly, the immobilization system employed in the treatment of Head and Neck Cancer (HNC) and Brain Cancer (BC) patients can pose challenges, as the widely accepted standard of care involves the use of a closed-faced thermoplastic mask (CFMs) [2,3]. This immobilization device, designed to cover the face only for BC treatments and also the neck and the shoulders for HNC, was developed with the aim of reducing set-up discrepancies [4,5]. If closed-face masks allow for a reproducible set-up, ensuring minimal head motion within the device [6,7,8] and a set-up uncertainty of only 2–3 mm [8,9], on the other hand, they may increase patients’ discomfort, pain, feelings of claustrophobia, and in some cases, even respiratory distress [1]. It has been widely reported that immobilization devices can play a pivotal role in triggering anxiety and affecting the emotional well-being of patients, thus representing one of the most distressing experiences during cancer treatment [10,11]. Up to 49% of HNC patients report distress and anxiety during the radiation course, with a major incidence before treatment starts [12]. A recent study by Nixon et al. recommended that routine screening for mask anxiety should be integrated throughout the course of RT, with the suggestion of multiple strategies to develop effective interventions in the management of mask anxiety [11].

Open-face masks (OFMs) are designed to leave certain parts of the face exposed, typically the forehead, the eyes, and the nose, as opposed to traditional radiotherapy masks that cover the entire face [5,13]. OFMs can promote patient comfort while ensuring the accuracy of immobilization and the reproducibility of effective positioning. The paradigm shift is completed by simultaneously exploiting the effect of optical systems capable of monitoring the exposed body surface and thus detecting all potential displacements. Hence, the inherent advantage of using such integrated monitoring systems with current Image-guided Radiotherapy (IGRT) systems is documented and confirmed for patients diagnosed with various diseases [5]. Moreover, open-face masks can be reinforced around the forehead and the chin symphysis to minimize potential longitudinal movement, thus providing adequate immobilization and ensuring greater comfort for claustrophobic patients [14].

The purpose of the present scoping review is to assess the current state of the art of open-face masks and to explore their potential use in clinical practice in the setting of HNC and BC patients through existing literature. Our review might indeed support their effectiveness in maintaining patient positioning and adherence to set-up without compromising treatment accuracy.

## 2. Materials and Methods

### 2.1. Design and Search Strategy

In accordance with the Preferred Reporting Items for Systematic Review and Meta-Analysis (PRISMA) extension for scoping reviews [15] and the Arskey and O’Malley framework [16], an electronic search was conducted to retrieve complete original studies reporting the use and description of OFMs in clinical practice. Studies were searched in EMBASE, PubMed, SCOPUS, and Web of Sciences until April 2024. The specific search strings for each database, developed and optimized with the support of the University of Florence library system, are provided in the “Supplementary Materials” section (Supplementary Table S1). The inclusion criteria were (a) English-language publications; (b) Studies addressing the use and/or the implementation of OFMs as an immobilization device for HNC and BC patients undergoing RT; (c) Studies performed on human subjects and/or phantoms. Studies were excluded if one of the domains for the inclusion criteria was not met. Conference abstracts, conference proceedings, and conference papers were excluded. According to the classification of types of medical research [17], both primary and secondary research studies published on the selected databases were included in the search strategy.

This literature search was performed by the Department of Allied Health Professions of Careggi University Hospital and by the Radiation Oncology Unit of Careggi University Hospital in collaboration with the Italian Association of Radiation Therapy and Medical Physics Technologists (AITRO).

A formal risk of bias assessment was deemed inappropriate for this scoping review, which is consistent with the framework established by Arksey and O’Malley [16].

### 2.2. Study Selection and Data Extraction

Following the literature search, all references were imported into reference management software (Mendeley Reference Manager v2.120.1). The software’s automatic duplicate detection function was used to identify and remove obvious duplicates. In addition, a manual screening was performed to ensure that all duplicates were correctly identified.

After the removal of duplicates, two independent reviewers (I.L. and N.I.) screened out irrelevant articles due to off-topic content by checking titles and abstracts. Subsequently, the full texts of eligible articles in question were then obtained and checked by the same reviewers for possible inclusion in the study. All discrepancies were resolved on a case-by-case discussion between the independent reviewers with the involvement of a third reviewer (C.V.).

Data were extracted by two reviewers (A.L., I.M.) and entered into an electronic database developed specifically for this scoping review.

The database made it easy to track, update, and export data for analysis. The final extracted data was reviewed by the entire research group to find any inconsistencies and ensure validity.

### 2.3. Analytic Approach

Following data extraction, the data were synthetized and discussed by the research team. After summarizing the study results, a standardized data extraction form in Microsoft Excel (Redmond, Washington) was used to consolidate the studies and to record key details such as authors, year of publication, type of study, sample size, treatment site, type of OFMs, aim, main findings and conclusions.

Ethical approval was not required for this study.

## 3. Results

### 3.1. Study Inclusion and Characteristics

A total of 19 articles were included in this study.

**Figure 1:**
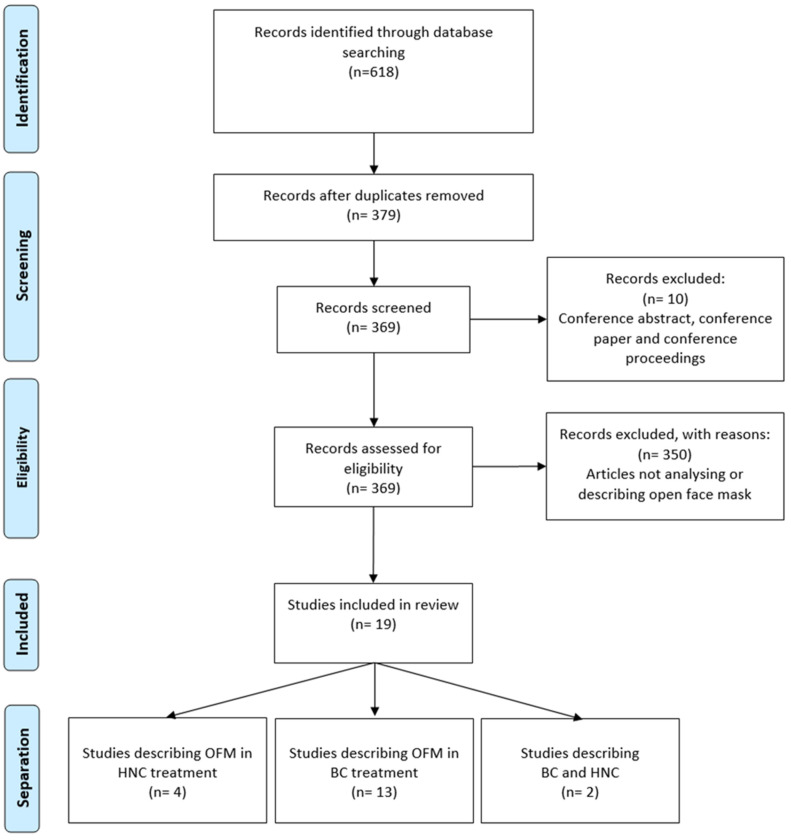
Flowchart of study selection.

Figure 1 shows the study selection process in the format of a PRISMA diagram.

All included articles were published between 2013 and 2024. Most of the studies were comparative (*n* = 13, 68.4%), whereas the others were observational studies (*n* = 6, 31.6%). Almost all studies (*n* = 18, 94.7%) were conducted on patients, except for one study that was conducted on anthropomorphic gel phantoms [18]. The sample size ranged from 7 to 269 patients.

**Figure 2:**
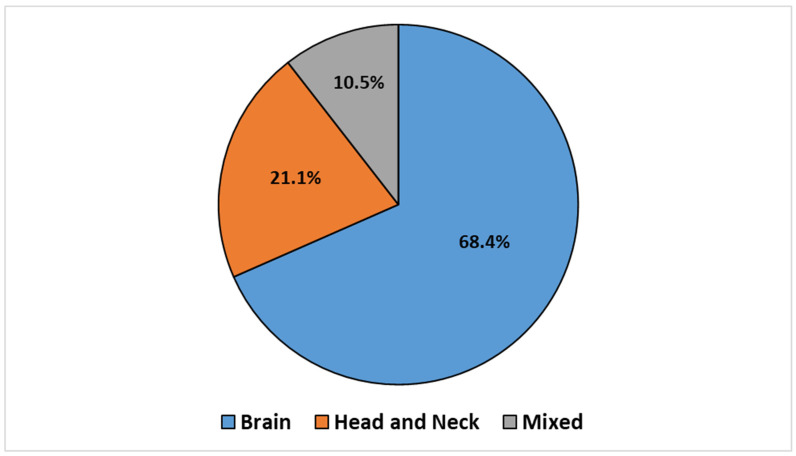
Retrieved articles stratified by treatment sites.

The articles were categorized according to the treatment site, which led to the identification of three groups: BC (*n* = 14, 68.4%), HNC (*n* = 4, 21.1%), and mixed sites (*n* = 2, 10.5%), which included both BC and HNC. This classification is illustrated in the pie chart reported in Figure 2. 

All articles included in the review were primary research studies (*n* = 19, 100%). An overview of the studies included in this scoping review is shown in Table 1.

### 3.2. Use of OFMs in BC Treatment

The adoption of OFMs in the RT setting for BC has been thoroughly investigated in 13 studies [13,18,19,20,21,22,23,24,25,26,27,28,29], along with two additional mixed-site studies [30,31]. Almost all articles were primarily focused on evaluating the precision and safety of OFMs in the delivery of Stereotactic Radiotherapy (SRT). Only one study focused on the use of OFMs in fractionated cranial Radiotherapy [28]. In all reported studies, the use of OFMs was systematically associated with the implementation of Surface-Guided Radiation Therapy (SGRT), except for the study by Ohira et al., where surface-guided patient set-up was not performed [29].

Four studies (26.6%) investigated the use of OFMs in combination with other devices to improve precision [19,24,27,30]. In this context, two studies investigated the use of OFMs in association with mouth bites to stabilize the head positioning during SRT [24,27]. Foster et al. [24] demonstrated that SGRT could detect intrafraction shifts greater than 1 mm in 50% of patients, which might have caused dosimetric deviations if uncorrected, thus highlighting the need for continuous monitoring and adaptation. Two other studies investigated the use of OFMS in combination with the Mayo head mold [19,30], a custom mold made in the CT simulation room that uses an expanding foam that conforms to the patient’s head, leaving its face uncovered [32]. Both studies showed that this combination could limit head motion within 1.5 mm during treatment [30] and provided sufficient accuracy to quickly set the patient up in the treatment room [19]. In the study by Lee et al., once the patient was positioned in the appropriate immobilization device, the head rotation was first corrected by adjusting the head position. As a result of the initial SGRT set-up, interfraction shifts measured with kV-Cone Beam Computed Tomography (kV-CBCT) were minimal, typically within 2 mm and 1° in any rotational axis [30].

Most studies (*n* = 12, 80.0%) reported that OFMs alone provided adequate immobilization with minimal impact on the precise delivery required for BC treatments [13,18,20,21,22,23,25,26,28,29,30,31]. Notably, these studies highlighted that OFMs achieved sub-millimeter accuracy (<1 mm) and sub-degree rotational precision (<1°) in intrafraction movements detected by SGRT, which are critical for the success of SRS protocols [18,20,22,23,25,28].

Among the identified studies, several (*n* = 5, 33.3%) evaluated the inter- and intrafraction movements of OFMs in a dual-shell or in a dual mask (anterior and posterior head mask) set-up, with excellent results in terms of stability and precision [13,21,22,26,29].

In line with the findings reported in the setting of HNC, a study by Keane et al. found that patient-reported comfort levels were higher with OFMs, meaning that patient discomfort was reduced without compromising positioning and immobilization accuracy [28].

### 3.3. Use of OFMs in HNC Treatment

The use of OFMs for the treatment of HNC patients was described in seven articles (five HNC studies [1,5,14,31,33] and two mixed [30,31]). All studies focused on the accuracy and safety of positioning using OFMs. In five studies (71.4%), the accuracy of OFMs set-up was evaluated in combination with SGRT to monitor and correct intrafraction motion [5,14,30,31,33]. In the study by Li et al. [14], the average three-dimensional (3D) Vector Length (VL) was 0.8 ± 0.3 mm and 0.4° ± 0.2°, while in the study by Wiant et al. [5] 0.9 ± 0.5 mm and 0.3°, data that overall supported the correct sub-millimeter OFMs immobilization and stability both in translations and rotations [14]. The study by Bry et al. focused on the accuracy of positional corrections detected by SGRT in four different facial expressions (open eyes, closed eyes, fear, and annoyance) within four Regions of Interest (ROIs) of different sizes on the face, which simulated possible mask openings, and two different spatial resolutions (standard resolution and SRS resolution) [31]. This study showed minimal baseline deviation for closed eyes (0.3 ± 0.3 mm), while expressions of fear and annoyance created greater false corrections that increased at standard resolution and smaller ROIs (2 ± 1.8 mm).

According to the study by Rudat et al., SGRT opened the possibility of reducing the number of CBCTs while maintaining sufficient set-up accuracy. The advantage was a reduction in imaging dose and overall treatment time [33].

Four studies (*n* = 4, 57.1%) compared inter-fraction motion between OFMs versus standard CFMs set-up [1,5,14,33]. The studies by Wiant et al., Mulla et al., and Rudat et al. compared translational and rotational inter-fraction shifts verifying Six Degrees of Freedom (6DOF) corrections based on the pre-treatment kV-CBCT [1,5,33]. The study by Rudat et al. verified daily patient set-up translations using an On-Board Imager (OBI) kV X-ray orthogonal pair imaging [33].

All these studies agreed on using OFMs to ensure immobilization and accuracy because measured differences were not clinically relevant: maximum observed 1 mm in the translational axis and <1° in the rotational axis.

In addition, two studies (*n* = 2, 28.6%) investigated patient comfort by comparing the OFMs with the standard CFMs set-up using a Likert-scale questionnaire [1,5]. In the study by Wiant et al. [5], the two groups did not differ significantly from each other, while in the study by Mulla et al. [1], patients reported higher neck and shoulder comfort and overall satisfaction when using the OFMs during CT simulation.

## 4. Discussion

The use of OFMs for HNC and BC was widely discussed in many recently published articles (82.4% from 2020 to the present, *n* = 16) with emphasis on the potential benefits in terms of patient comfort, increased treatment accuracy, and effective monitoring of intrafraction movement with SGRT technology.

This review comprehensively examined different studies investigating the feasibility, efficacy, and challenges associated with OFMs at different anatomical sites. Of note, one-third of the included articles (*n* = 8, 33.3%) compared OFMs with CFMs in BC and HNC [1,5,13,14,27,28,29,33]. The selected articles were very heterogeneous. Some studies indeed compared different immobilization devices, while others focused on the use of SGRT technology to monitor inter- and intrafraction motion. Despite the heterogeneity of the themes addressed, all the included articles included the topic of set-up accuracy.

The use of OFMs in RT for BC treatment was extensively evaluated in 13 studies where two consistent key points were highlighted: the use of OFM devices in SRS and SRT treatments and the pivotal role of SGRT in addition to OFMs in delivering RT. In this context, various types of immobilization devices could be adopted; OFMs could be used indeed either alone [13,18,20,21,22,23,25,28,29,30,31] or with complementary immobilization devices, such as a mouthbites [24,27] or with dual shell or dual mask [13,21,22,26,29] (anterior and posterior) or Accuform Cushion [22], instead of traditional headrest, or again with Mayo Mold [19,30]. The analysis indicated that various immobilization devices could be employed for BC treatment.

These systems differed not only in the mask’s opening, with smaller or larger areas exposed but also in the use of complementary equipment related to patient positioning. Due to the high heterogeneity of devices used in the included articles, it is challenging to compare studies regarding interfraction or intrafraction motion. However, the wide range of available OFMs and complementary devices allows RT departments to choose the immobilization devices that best meet their needs and those of the patient, emphasizing increasingly personalized RT and ensuring reproducibility with the use of appropriate complementary devices.

Overall, regarding the interfraction shifts in BC, all studies agreed on the stability and reproducibility of OFMs within 2 mm in every translation axis and 1° in every rotational axis [22,27,28]. The measurement of intrafraction variability as well was reported in several articles, and the findings consistently agreed on the reproducibility, highlighting that OFMs limited head motion to within 1 mm and 1° [13,21,22,25,28,29].

Regarding the utilization of OFMs in HNC treatments, six studies were included and analyzed. Several studies compared OFMs with CFMs, and their findings, consistent across the evidence, indicated that OFMs provided similar set-up accuracy and ensured adequate immobilization comparable to CFMs [1,5,33].

In addition to precision and accuracy, patient comfort is a critical factor in RT. In this regard, many studies comparing OFMs with CFMs emphasized the importance of using OFMs in clinical practice to improve patient comfort. In particular, the study by Li et al. included a survey of healthy volunteers on the comfort they experienced with both masks [14]. The results showed that 80% preferred the OFMs because the CFMs required the subjects to keep their eyes and mouth closed during treatment. The study by Wiant et al. also used a survey method to assess patient comfort and found that the mean scores for anxiety, claustrophobia, and drug use were lower in the OFMs group [5]. Consistent results were confirmed in the study by Mulla et al., where patients treated with OFMs reported feeling less tightness and anxiety and were overall more satisfied and comfortable in the neck and shoulder area than patients treated with CFMs [1]. Regarding the assessment of patient comfort in the BC setting, its evaluation was not the primary focus for most studies, whereas technical and clinical feasibility were further explored [22,29,30]. Patient comfort was widely investigated only in the study by Keane et al. [28], where the use of OFM systems was associated with a significant improvement in patient comfort, allowing a reduction in anxiety and pain without compromising the accuracy of immobilization and patient set-up.

Since OFMs and CFMs are made of the same material and fabricated in the same way, no additional training is required for the use of OFMs in clinical practice and the costs differ only minimally [32].

Another key point across several studies was the integration of the SGRT system with OFMs in clinical practice for both treatment sites. According to most studies, OFMs enabled the integration of SGRT for both interfraction displacement detection and intrafraction motion monitoring [18,20,22,24,25,26,27,30,33]. In addition, the use of SGRT improved the set-up accuracy for non-coplanar treatment, that involved complex positions and angles [20,27]. In the study by Gregucci et al., it is reported that the combination of OFMs and SGRT could potentially replace conventional radiological imaging, such as IGRT two-dimensional (2D) or 3D, in evaluating patient positioning in conventional RT for BC [22]. In addition, one of the potential benefits of integrating the SGRT system with OFMs is the reduction in patient set-up time, as reported by Lee et al. [23], where the pure SGRT set-up time, including patient positioning, was less than 1 min (0.8 ± 0.3 min).

On the other hand, a limitation of SGRT was reported in the study by Bry et al., which found that the SGRT system could generate wrong position corrections according to different patients’ facial expressions [31]. This limitation was particularly evident when lower ROIs and lower resolution were used on the SGRT system.

### 4.1. Recommendations for Clinical Practice

Several recommendations for the use of OFMs in clinical practice can be derived from the comprehensive analysis of the study results. These recommendations are intended to guide healthcare professionals in optimizing the implementation of OFMs and ensure their effective integration into clinical workflows:

- During the simulation phase: customized OFMs are built, and additional stabilizing devices are used. For patients who require enhanced stabilization, the use of additional devices such as mouth bites or custom molds like the Mayo head mold can be beneficial. These systems, when used in combination with OFMs, can further limit head motion and improve stability and precision;
- During daily RT: a combination of SGRT and IGRT technologies are used to correct patient positioning and detect intrafraction motion. After the initial positioning of the patient by means of immobilization devices, the SGRT system is used to assess the set-up and minimize interfraction shifts. IGRT is then used to assess and confirm patient positioning. Once all necessary corrections have been made, the SGRT system plays a central role in detecting intrafraction movements. Continuous monitoring of patient positioning is crucial to detect and correct any displacements that occur during RT. The role of SGRT is essential for the effective use of OFMs in clinical practice, but despite its use, IGRT remains essential, especially for hypofractionated treatments. The possibility of reducing the frequency of IGRT was only suggested in the study by Gregucci et al. for conventional RT in BC treatments [22].

**Figure 3:**
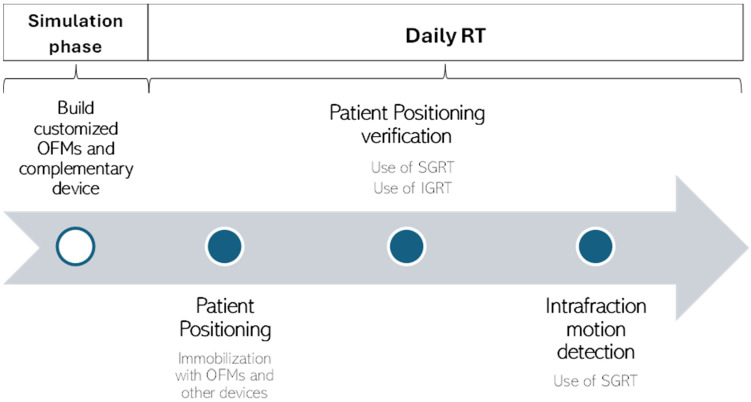
A workflow for implementing OFMs in clinical practice based on recommendations from the included studies.

Based on these recommendations, we developed a workflow (Figure 3) for the implementation of OFMs in clinical practice. This workflow outlines the specific phases in which SGRT and IGRT play a role in the RT workflow.

### 4.2. Limitations

Some limitations of the present scoping review and of the included studies must be outlined. Firstly, all studies were single-institution studies, which means that the results may not be generalizable due to the unique clinical setting and protocols of each institution. Also, results related to set-up accuracy may not be widely applicable as they could be influenced by the specific type of OFMs used in clinical practice and by the clinical experience and expertise of the treatment team, as referred to in the study by Han et al. [25]. Secondly, many of these studies utilized a retrospective design, which inherently introduces biases and limitations associated with such methods [33]. Finally, some evidence, such as the one by Zhou et al. [26], is based on initial clinical experience with new emerging techniques or technologies, such as OFMs immobilization in combination with the SGRT system. This early implementation phase may further limit the applicability and robustness of the results, suggesting that the workflow designed for the implementation of SGRT and OFMs can be further optimized. Regarding the limitations of the scoping review itself, only a narrative synthesis method was conducted in reporting the findings, with no clear method for quantitatively synthetizing the results. Also, another noted limitation is that only studies published in English were included.

## 5. Conclusions

The use of OFMs with the integration of SGRT for BC and HNC offers significant advantages in terms of patient comfort and accuracy. The included studies consistently demonstrated that sub-millimeter and sub-degree reproducibility of patient positioning can be achieved with OFMs, particularly when combined with the SGRT system and other complementary devices. These results support the use of OFMs in clinical practice to enhance the precision of RT while improving patient comfort when compared to CFMs. Future research should further explore innovative combinations of immobilization devices and monitoring technologies to continuously improve RT outcomes.

## Figures and Tables

**Table 1:** Summary of main findings from studies on the use of open-face masks in brain and head and neck cancer treatments.

First Author and Year	Type of the Study	Sample Size	Treatment Site	Aim	Type of OFMs	Main Findings	Conclusions
Li et al. (2013) [14]	Comparative study	15 (10 healthy volunteers and 5 claustrophobic patients)	HNC	Evaluate quantitatively the immobilization performance of the OFM.	3 points OFM (Orfit Industrie)	The OFMs did not reduce the strength of the mask and provided immobilization within 2.0 mm with improved comfort and tolerability. The average motion of claustrophobic patients was similar to that experienced by volunteers.	The study demonstrated that the greater tolerability of the OFM may allow its use in a larger population.
Li et al. (2015) [19]	Comparative study	33 (25 PinPoint vs. 8 Freedom)	BC	Compare two clinical immobilization systems (Freedom vs. PinPoint) for cranial fSRS.	Mayo head mold with OFM using the Freedom immobilization system	Both intracranial fSRS immobilization systems could restrict head motion within 1.5 mm during treatment as monitored by OSI. Freedom system outperformed the PinPoint system in terms of patient comfort and clinical workflow.	The study underscored the advantages of the Freedom system in intracranial fSRS due to its superior motion control, set-up accuracy, patient comfort, and clinical workflow.
Wiant et al. (2016) [5]	Prospective Comparative study	50 (25 CFMs vs. 25 OFMs)	HNC	Comparison between the use of OFMs and CFMs for HNC RT.	Open thermoplastic head and shoulder mask Openview Assure (Qfix)	OFMs effectively limited motion comparably to CFMs across treatment up to 35 fractions. The OFMs group showed reduced mean values of anxiety, claustrophobia, and drug use, but none of these distributions were significantly different.	OFMs provided comparable immobilization and posture preservation to CFMs for HNC RT.
Mulla et al. (2020) [1]	Prospective Comparative study	40 (20 CFMs vs. 20 OFMs)	HNC	Determine the set-up reproducibility and level of comfort and satisfaction in the patients immobilized with OFMs versus CFMs.	Duon closed head and shoulder mask (Orfit); 5-point hybrid head and shoulder mask (Orfit) with AccuForm Cushion	Comparison between CFMs and OFMs patients showed similar set-up accuracy, based on translational and rotational shifts, ensuring adequate immobilization without compromising patient comfort. Patients reported higher satisfaction with OFMs due to reduced feelings of tightness and anxiety compared to CFMs.	OFMs provided comparable yet comfortable immobilization to CFMs for HN RT.
Swinnen et al. (2020) [20]	Observational study	7	BC	Demonstrate the implementation of the SGRT system for intrafraction motion during treatment for a non-coplanar VMAT technique.	3 points OFMS (Orfit Industrie) and T-shaped vacuum bag	SGRT system could significantly improve the set-up accuracy for treatment involving complex angles and positions.	The integration of SGRT systems with OFMs in a non-coplanar single isocenter framework was feasible and effective for high-precision SRS of brain metastases.
De Ornelas et al. (2021) [21]	Observational study	95	BC	Evaluate intra-fraction target shift during automated mono-isocentric linac-based SRS with OFMs system and optical real-time tracking.	Encompass mask (double-mask system) (Qfix)	Intra-fraction motion in SRS treatment required an additional margin to the PTV. A 1 mm PTV margin was insufficient in 18% of targets at a distance greater than 6 cm away from the isocenter but sufficient for 96% of targets within 6 cm.	A PTV expansion of 1 mm was recommended due to intra-fractional movement to ensure target coverage for planes with isocentric positioning less than 6 cm away from targets.
Gregucci et al. (2021) [22]	Prospective observational study	69 (24 cRT; 45 SRT)	BC	Evaluate inter-fraction reproducibility, intrafraction stability, technician aspects, and patient/physician’s comfort of OFMs cranial RT.	Solstice system (dual shell) with OFM and Accuform Cushion	The inter-fraction CBCT mean values were analyzed in all translational and rotational directions, and it was found that motion was <1 mm and <1°, respectively. The analyses of the intrafraction CBCT showed that the translational values were <0.05 mm and the rotational values were <0.5°.	The proposed immobilization solution allowed the use of 1 mm CTV-PTV margin for Linac-based SRT. With OFMs and SGRT, radiological imaging could be omitted for cRT.
Lee SK et al. (2021) [23]	Retrospective Comparative study	269	BC HNC	Evaluate the accuracy of SGRT in cranial patient set-up compared to CBCT shifts.	OFM with CDR head immobilization device	The SGRT set-up difference (magnitude) compared to CBCT shifts, was 1.0 ± 2.5 mm and 0.1° ± 1.4°. The SGRT set-up time was much shorter than that of CBCT and 2 DkV set-ups.	The SGRT system had sufficient accuracy to quickly set the patient up and enabled real-time motion monitoring of BC and nasopharynx cancer patients immobilized with OFMs.
Bry et al. (2022) [24]	Prospective study	10 healthy human subjects	BC HNC	Quantify the false positional corrections produced by the SGRT system due to face motion in a patient immobilized with OFMs.	Orfit’s 3-point OFM; Brainlab’s SRS immobilization mask	The average deviation observed due to changing facial expressions was 1.4 ± 0.9 mm for SRS-specific and 1.6 ± 1.6 mm for standard resolution. Position corrections in the SGRT system could be affected by the patient’s facial expressions.	False corrections in an SGRT system due to different facial expressions should be considered during treatment planning.
Bry et al. (2022) [18]	Comparative study	N/A	BC	Verify the positioning accuracy of an SGRT system compared to X-ray imaging in a phantom positioned with OFMs.	OFM	Discrepancies between the SGRT combined with the OFM and stereoscopic X-ray set-ups were less than 1 mm in translation and less than 0.5 degrees in rotation. Surface imaging demonstrated high accuracy and reproducibility comparable to X-ray imaging for position verification in SRS treatments.	SGRT was feasible for position verification in SRS, showing accuracy and reproducibility comparable to orthogonal X-ray imaging.
Da Silva et al. (2022) [25]	Comparative study	14	BC	Provide information about the geometric accuracy of EXTD and the application of its workflow in clinical practice.	cranial 4Pi OFM Brainlab	The combination of optical/thermal and stereoscopic X-ray technology achieved sub-millimeter accuracy in alignment with CBCT, demonstrating high geometric precision in patient set-up. The EXTD system, incorporating an OFM with SGRT and IGRT modalities, allowed for continuous and real-time patient monitoring and positioning with high accuracy.	The EXTD system provided an accurate and reliable method for patient positioning in RT, particularly useful in treatments requiring high precision such as SRS treatment.
Foster et al. (2022) [26]	Retrospective study	55	BC	Investigate the dosimetric consequences of uncorrected intrafraction patient motion detected during frameless linac-based SRS, immobilized with an OFMs	Encompass mask with biteplate (QFix)	In 25 patients, SGRT detected ≥1 mm shifts, indicating potential GTV underdosages and increased healthy brain doses if uncorrected. The treatment technique (cone vs. MLC) influenced the robustness of the plan against motion.	SGRT detected intra-fraction motion in frameless SRS, leading to underdosages and increased normal brain doses.
Han et al. (2022) [27]	Retrospective Comparative study	21 (10 PinPoint vs. 11 OFMs)	BC	Compare intrafractional motion using two different immobilization systems (Aktina PinPoint and vacuum-suction customized mouthpiece vs. OFMs) under the guidance of an SGRT system.	OFM (Klarity Medical Products)	Patients immobilized with the OFMs system showed a significantly greater variation in intrafraction movement in both translations and rotations than patients immobilized with a vacuum fixation biteplate. Both the vacuum fixation system and the OFMs system limited intrafraction rotations.	In patients with the vacuum fixation system, the intrafraction motion variation was significantly lower than in patients with the OFMs. The SGRT is recommended to minimize intrafraction motion.
Ohira et al. (2022) [28]	Comparative study	76 (38 CFMs vs. OFMs)	BC	Compare the intrafraction motion during the cranial SRT in patients immobilized with OFMs and CFMs.	Encompass mask (double-mask system) (Qfix)	No statistically significant difference was observed between the intrafraction motion of the two immobilization devices in translational and rotational axes, except in the anterior–posterior direction (*p* = 0.02). The margin compensation for intrafraction motion was less than 1 mm for both immobilization devices.	The intrafraction motion in SRS using OFMs and CFMs was approximately equal considering the adequate accuracy in patient positioning.
Reitz et al. (2022) [13]	Comparative study	40	BC	Compare the magnitudes of intrafraction deviation for four different mask systems.	iCAST Head Double Micro OFMS (IT-V); Cranial 4Pi OFM Brainlab; Brainlab stereotactic mask	The results showed deviations lower than 0.6 mm and 0.6° when using one of the four thermoplastic mask systems; outliers with a translational deviation of more than one millimeter can occur with OFMs systems.	Deviations were smaller than 0.6 mm in all translation directions and smaller than 0.6° in all rotation axes using 4 different thermoplastic mask systems with IGRT.
Zhou et al. (2022) [29]	Retrospective study	48	BC	Propose a dedicated surface-guided SRT treatment procedure with OFMs immobilization and evaluate the initial clinical experience to improve set-up accuracy.	Open-face double shell (MacroMedics)	The treatment procedure was reasonably efficient for routine clinical use, with minimized initial set-up errors and a low repositioning rate. SGRT was a complement to CBCT and not an alternative that could replace it.	The proposed surface-guided SRT procedure with OFMs immobilization was a step forward in improving patient comfort and positioning accuracy in the same process.
Chen et al. (2023) [30]	Comparative study	40 (20 CFMs vs. 20 OFMs)	BC	Evaluate the accuracy, reliability, and feasibility of using SGRT for positioning guidance in patients immobilized with an OFMs and mouth bite device	OFM combined with mouth bite (Klarity)	The OFMs group achieved superior positioning accuracy in SRT, with significantly lower translation and rotation errors compared to the CFMs group, enhancing precision and reducing set-up errors.	The OFMs and mouth bite enhanced precision and stability in brain SRT using SGRT technology.
Rudat et al. (2023) [31]	Comparative study	44 (13 CFMs vs. 31 OFMs)	HNC	Compare the set-up accuracy of patients positioned using SGRT with patients positioned using in-room laser alignment with patient skin marks	OFM	Comparable set-up margins were found for OFMs with the SGRT system compared to CFMs with laser alignment and mask marks. Given the low set-up error when using OFMs, SGRT in the HNC was more relevant when higher doses needed to be administered.	OFMs may be used instead of CFMs to increase patients’ comfort.
Keane et al. (2024) [32]	Comparative study	30	BC	Compare patient discomfort and immobilization performance of OFMs and CFMs in cranial radiotherapy	Five-point OFMS Clear Vision 2 (CIVCO Radiotherapy)	OFMs significantly reduced discomfort, anxiety, and pain (*p* < 0.0001 for all) compared to CFMs. While CFMs showed smaller interfraction longitudinal displacements, roll, and yaw rotations (*p* < 0.05), they showed larger lateral displacements compared to OFMs combined with an SGRT system. Intrafraction variability did not differ between the masks.	OFMs were associated with decreased patient discomfort without compromising patient positioning and immobilization accuracy.

AP: Anterior-Posterior; BC: Brain Cancer; CBCT: Cone Beam Computed Tomography; CFMs: Closed Face-Masks; CHR: customized headrest; cRT: Conventional Radiotherapy; CTV: Clinical Target Volume; EXTD: ExacTrac Dynamic; fSRT: frameless stereotactic radiosurgery; GTV: Gross Tumor Volume; HNC: Head and Neck Cancer; IGRT: Image Guided Radiotherapy; MLC: Multileaf Collimator; OFMs: Open-Face Masks; OSI: Optical Surface Imaging; PTV: Planning Target Volume; ROI: Region of interest; RT: Radiotherapy; SGRT: Surface Guided RadioTherapy; SRS: Stereotactic Radiosurgery; SRT: Stereotactic Radiotherapy; VMAT: Volumetric Modulated Arc Therapy; WL: Winston–Lutz test.

## Supplementary Materials

The following supporting information can be downloaded at https://www.mdpi.com/article/10.3390/cancers16162899/s1, Table S1: Full search strings for each database.
