# Supplementary material for: Open-Face Masks in Radiotherapy: Enhancing Therapeutic Strategies for Head and Neck and Brain Cancer Patients—A Comprehensive Scoping Review
Source: Cancers (Basel). 2024 Aug 21;16(16):2899. doi: 10.3390/cancers16162899 (PMC11353183; doi:10.3390/cancers16162899)
Supplement: Supplementary file 1 [file cancers-16-02899-s001.zip › cancers-3162444-supplementary.pdf]

Supplementary table S1. Full search strings for each database

Database	Search string
PubMed	(("Head and Neck Neoplasms"[MeSH Terms] AND "Masks"[MeSH Terms]) OR (("open face mask*"[Title/Abstract] OR "open mask*"[Title/Abstract] OR ("open face"[Title/Abstract] AND ("head"[MeSH Terms] OR "head"[All Fields] OR ("neck"[MeSH Terms] OR "neck"[All Fields]) OR "radio*"[All Fields] OR ("brain"[MeSH Terms] OR "brain"[All Fields] OR "brains"[All Fields] OR "brain s"[All Fields]) OR ("cerebrally"[All Fields] OR "cerebrum"[MeSH Terms] OR "cerebrum"[All Fields] OR "cerebral"[All Fields] OR "brain"[MeSH Terms] OR "brain"[All Fields])))) AND 2000/01/01:2024/12/31[Date - Publication])) AND (2000:2024[pdat])
EMBASE	('head and neck tumor'/exp AND 'face mask'/exp) OR ('open face mask':ti,ab OR 'open face masks':ti,ab OR 'open mask':ti,ab OR 'open masks':ti,ab OR ('open face':ti,ab AND ('head'/exp OR head OR 'neck'/exp OR neck OR 'radio*')) AND ([article]/lim OR [article in press]/lim OR [data papers]/lim OR [editorial]/lim OR [letter]/lim OR [note]/lim OR [review]/lim) AND [2000-2024]/py
Web Of Science	((TI=("open face mask" OR "open face masks" OR "open mask" OR "open masks" OR ("open face" AND (head OR neck OR radio*))))<br>OR<br>AB=("open face mask" OR "open face masks" OR "open mask" OR "open masks" OR ("open face" AND (head OR neck OR radio*))))<br>NOT (DT==("PROCEEDINGS PAPER" OR "MEETING ABSTRACT" OR "RETRACTED PUBLICATION"))<br>Filter:2000-2024
SCOPUS	(TITLE-ABS ("open face mask" OR "open face masks" OR "open mask" OR "open masks" OR ("open face" AND (head OR neck OR radio*))))<br>OR<br>(INDEXTERMS (("Head and Neck Neoplasms" AND "Masks") OR ('head AND neck AND tumor' AND ''face AND mask')))<br>AND<br>(LIMIT-TO (DOCTYPE , "ar") OR LIMIT-TO (DOCTYPE , "re") OR LIMIT-TO (DOCTYPE , "sh") OR EXCLUDE (DOCTYPE , "cp"))<br>AND<br>date of publication: 2000-2024
